# How does artificial intelligence empower EFL teaching and learning nowadays? A review on artificial intelligence in the EFL context

**DOI:** 10.3389/fpsyg.2022.1049401

**Published:** 2022-11-16

**Authors:** Ruihong Jiang

**Affiliations:** College of Humanities and Arts, Tianjin University of Finance and Economics Pearl River College, Tianjin, China

**Keywords:** artificial intelligence, AI in the EFL context, affective computing, English as foreign language (EFL), EFL teaching and learning

## Abstract

The booming Artificial Intelligence (AI) provides fertile ground for AI in education. So far, few reviews have been deployed to explore how AI empowers English as Foreign Language (EFL) teaching and learning. This study attempts to give a brief yet profound overview of AI in the EFL context by summarizing and delineating six dominant forms of AI application, including Automatic Evaluation Systems, Neural Machine Translation Tools, Intelligent Tutoring Systems (ITSs), AI Chatting Robots, Intelligent Virtual Environment, and Affective Computing (AC) in ITSs. The review furthermore uncovers a current paucity of research on applying AC in the EFL context and exploring pedagogical and ethical implications of AI in the EFL context. Ultimately, challenges from technical and teachers' perspectives, as well as future research directions, are illuminated, hopefully proffering new insights for the future study.

## Introduction

With Artificial Intelligence (AI) consistently penetrating all domains of human life, recent years have witnessed the dramatic growth of AI in education (AIEd) (Hwang et al., 2020). Specifically, AIEd is anticipated to grow by 43% from 2018 to 2022 (Becker et al., 2018), whereas the Horizon Report 2019 (Alexander et al., 2019) predicts AI applications of teaching and learning will develop even more significantly. As one key issue discussed by UNESCO (2019), AIEd proliferates surging scientific output (Hinojo-Lucena et al., 2019). Likewise, various AI applications integrating analytical techniques [e.g., Machine Learning (ML), Natural Language Processing (NLP), Artificial Neural Networks (ANNs), Affective Computing (AC)], have been widely harnessed in English as Foreign Language (EFL) context and are exerting profound impacts. Particularly in the upheaval of teaching modes in the COVID-19 pandemic, video conferencing tools (e.g., Zoom) and learning management systems (e.g., Blackboard) supported by AI technologies have been widely opted to implement online EFL teaching and learning (Layali and Al-Shlowiy, 2020). A large and growing body of literature has suggested that AI can benefit language teaching and learning (Gao, 2021; Pikhart, 2021; Klimova et al., 2022), and ameliorate the quality of online EFL learning especially during the COVID-19 pandemic (Zitouni, 2022). To date, many reviews on AIEd have emerged (Chen et al., 2022). Nevertheless, to our knowledge, reviews concentrating on AI in the EFL context tend to be comparatively scarce. Accordingly, this paper attempts to explore how AI empowers EFL teaching and learning nowadays through critically reviewing research into the application of AI technologies solely in the EFL context, hopefully inspiring more fresh ideas in this future domain.

An automated method (Guan et al., 2020) was used to initially retrieve related publications from 2000 to 2022 in four databases (i.e., WOS, Scopus, ERIC, and Google Scholar) by searching AI-related terms (e.g., Chatting Robots) and EFL-related terms (e.g., EFL/English Teaching and Learning). Afterwards, the publication relevance was assessed by experts (Chen et al., 2022) to determine targeted research samples in this review. Manual coding and keyword analysis, along with computer-assisted content analysis (i.e., NVivo), were deployed to profoundly analyze the highly-related publications (*N* = 245). Consequently, the following six dominant forms of AI in the EFL context were summarized, including Automatic Evaluation Systems, Neural Machine Translation Tools, Intelligent Tutoring Systems, AI Chatting Robots, Intelligent Virtual Environment, and Affective Computing in ITSs. Moreover, this review teased out AI in the EFL context in the literature from learner-facing and teacher-facing perspectives. Finally, the paper endeavors to shed new light on challenges from technical and teachers' perspectives and future research directions of AI in the EFL context.

## Automatic evaluation systems

Automatic Evaluation Systems (AESs) are generally based on big data and NLP technologies (e.g., automatic speech recognition, word sense disambiguation, etc.) to evaluate the input information and provide automatic revision opinions, which are mainly applied in EFL writing and speaking contexts. Current commercialized applications using AESs in EFL writing, namely Criterion and Pigai, are proved to bolster writing accuracy and motivate learners to do more writing practice and revision (Bai and Hu, 2017; Gao, 2021). Likewise, AESs in EFL speaking, such as English 60 Junior and Eye speak, are confirmed to improve oral proficiency, frequency, and pronunciation (Ahn and Lee, 2016). In the covid-19 context, AESs can also facilitate EFL teaching by integrating online evaluation with automatic response and grading (Zitouni, 2022). However, from teachers' perception, they may remain vaguely optimistic about the use of AESs. Some underscore that AESs can't replace human raters in EFL writing instruction (Bai and Hu, 2017; Qian et al., 2021) due to relatively low accuracy (Liu and Kunnan, 2016), insufficient high-quality comment on collocation errors and syntactic use (Gao, 2021), the frustrating levels of recognition, and the lack of convenience (McCrocklin, 2019). Since teachers' adoption of AI is determined by its effectiveness and efficiency (Du and Gao, 2022), from technical perspectives, more technical efforts need to be exerted to improve the assessment accuracy of AESs; from instructional perspectives, teachers are required to fully deploy the potential of AESs to improve their effectiveness in diverse EFL contexts.

## Neural machine translation tools

Neural Machine Translation (NMT) is an end-to-end learning approach for automated translation. Unlike the traditional statistical MT, NMT aims to build a single neural network to maximize translation performance and overcome many weaknesses of conventional phrase-based translation systems (Bahdanau et al., 2014; Wu et al., 2016b). Google Translate, the Microsoft Translator (Bing), etc., are nowadays widely used NMT tools in diverse language education, of which performance is evaluated and compared from different linguistics perspectives (Vanjani and Aiken, 2020; Koh, 2022), still unveiling pressing problems, for instance, being incapable of conveying the proper sense and implication at the discourse level, containing errors of discontinuous expressions, word orders, etc., and requiring humans' intensive post-editing (Groves and Mundt, 2015; Koh, 2022). Notwithstanding the drawbacks of NMT tools revealed in some literature (Bahri and Mahadi, 2016), a considerable amount of literature suggest that NMT tools can benefit EFL students' learning: from the cognitive and linguistic perspective, they promote self-directed learning (Godwin-Jones, 2015), improve mastery of lexico-grammatical knowledge (Doherty and Kenny, 2014; Bahri and Mahadi, 2016), and develop productive (L2 writing) and receptive (reading comprehension) language skills (Alhaisoni and Alhaysony, 2017). It is noteworthy that from the affective perspective, they may lower language anxiety (Bahri and Mahadi, 2016). Conversely, Zhu et al. (2020) recently found that NMT may inhibit learning motivation. Moreover, some research reveals that NMT tools are especially suitable for advanced L2 learners (Klimova et al., 2022). Pertaining to NMT tools' pedagogical implications, they can be exploited to conduct comparison activities of original and machine-translated texts (Chen et al., 2020) and engage students in analytical language tasks or awareness-raising tasks (Valijärvi and Tarsoly, 2019). Overall, the use of MNT tools in the EFL context remains controversial, especially in the teaching context (Delorme Benites and Lehr, 2022). Future research can focus on the following two unanswered questions: considering EFL learners, whether NMT tools can benefit all English proficiency levels require more empirical research to investigate thoroughly; for EFL teachers, how to effectively exploit NMT tools to maximize their effectiveness and adaptability in EFL classroom requires more in-depth exploration.

## Intelligent tutoring systems

As computer-based learning systems, Intelligent Tutoring Systems (ITSs) are designed to promote personal tutoring and facilitate learning based on learner models, algorithms, and neural networks. Through providing proper and immediate feedback and tailoring instructional materials, various applications of ITSs, such as Pigaiwang (for Chinese students English writing), Your Verbal Zone (for Turkish students English vocabulary learning), and Robo-Sensei (for Japanese), are widely adopted in EFL fields, which have yielded learning effectiveness, for instance, improving grammar learning (Abu Ghali et al., 2018) and reading comprehension (Xu et al., 2019). ITSs can also be adapted to various teaching contexts to maximize their effectiveness. Concretely, embedded in the flipped classroom, ITSs help students solve problems (Mohamed and Lamia, 2018); assisting Self-Regulatory Learning (SRL), ITSs are proved to improve speaking skills (Mohammadzadeh and Sarkhosh, 2018); in COVID-19 context, ITSs can build students' performance profiles to assist teachers in adapting online teaching modes and content wisely (Nagro, 2021). Surprisingly, few attempts have been made to detect EFL learners' affective state when applying ITSs, although Affective Computing (AC) has been widely embedded in ITSs in other education domains. Hence, it may be pedagogically significant to integrate AC into current ITSs in the EFL context to identify and classify learners' emotions and give adequate emotional support to motivate EFL learning.

## AI chatting robots

AI Chatting Robots (AI chatbots) are computer programs with AI to promote intelligent human language interaction in a written or spoken form, which can provide a more fluid user experience by updating their knowledge and perception from previous conversations (Haristiani, 2019). Many empirical studies have confirmed the effectiveness of AI Chatbots in EFL fields. Concretely, AI Chatbots can not only strengthen EFL learners' mastery of language knowledge, including grammar and new vocabularies (Wang and Petrina, 2013), but also can improve English application skills, namely oral communication skills, listening and reading skills, and high-quality argumentative writing skills (Hong et al., 2016; Kim et al., 2019; Guo et al., 2022). Additionally, some research reveals that AI Chatbots can boost students' motivation, self-confidence, and interest in learning (Kim et al., 2019). However, whether AI Chatbots benefit all English proficiency levels remains controversial: some research argues that chatbots are ineffective for beginners, while some claim that all students can benefit (Kim, 2016). Additionally, it's noteworthy that some minor pronunciation errors along with grammar and spelling mistakes seem challenging for present AI Chatbots to interpret and diagnose in EFL fields. As Lotze (2018) argues, AI diagnostic systems still need to meet some key criteria (i.e., spontaneity, creativity, and shared knowledge) before they can serve as a real-life language teacher. Hence, along with providing more technical support to upgrade diagnostic systems of AI chatbots, future work is required to empirically investigate their adaptability to different language proficiency levels and various instructional contexts.

## Intelligent virtual environment

It is during the last two decades that Virtual Reality (VR) tools have been widely adopted in foreign language education (Rau et al., 2018; Wang et al., 2020), exemplified by Google Earth (Chen et al., 2020), Google Tour Creator (Nobrega and Rozenfeld, 2019), and Google Expeditions (Xie et al., 2021). Multiple benefits of applying VR tools in the EFL context have also been validated, for instance, improving vocabulary learning and retention (Lai and Chen, 2021; Tai et al., 2022), enhancing English speaking and willingness to communicate (Ebadi and Ebadijalal, 2020), building ideal 2L self (Adolphs et al., 2018), and improving English learning motivation to reduce anxiety (Chien et al., 2020). With AI technologies maturing enough to interact with a virtual environment, Intelligent Virtual Environment (IVE) is proposed as “a combination of intelligent techniques and tools, embodied in autonomous creatures and agents, together with effective means for their graphical representation and interaction of various kinds” (Luck and Aylett, 2000). As one typical application of IVE, virtual agents (avatars) can upgrade user presence in the virtual and promote collaboration (Yin, 2022). Particularly in the EFL context, 3D avatars are reported to improve listening performance (Lan et al., 2018). Similarly, some empirical evidence suggests that using avatars can decrease foreign language anxiety and encourage learners to communicate more successfully (Melchor-Couto, 2017; York et al., 2021). IVR can also serve as an effective means to engage EFL students virtually in Zoom virtual classroom teaching in covid-19 pandemic (Obari, 2020). However, some scholars still express some reservations about the effectiveness of using avatars in the EFL context, considering technical factors such as interacting with avatars outside the scripted application areas (Lotze, 2018), the acceptability of virtual avatars (Repetto, 2014). Additionally, the affordability and limited access to networks still stump the implementation of IVE in the EFL context (Cowie and Alizadeh, 2022). Thus, there is a call for more new technological frontiers to address the aforementioned technical problems, along with an appeal for EFL teachers' professional training to exploit IVE and avatars properly from technical and pedagogical aspects.

## Affective computing in ITSs

There is a consensus that emotion significantly impacts cognitive activities (e.g., learning) (Wu et al., 2016a, 2022; Ma and Lin, 2017). Particularly, the close connection between emotions and language learning motivation is also empirically verified (Yu et al., 2022). Affective computing (AC) is currently one of the most promising research topics (Tao and Tan, 2005), referring to “computing that relates to, arises from, or deliberately influences emotion or other affective phenomena” (Picard, 1997). By recognizing learners' emotions from physiological, facial, and textual data, AC is widely embodied in ITSs and applied in various educational domains. To illustrate, the Affective Tutoring System (ATS) combining emotional expressions and AC, can detect learners' emotional expressions, determine learners' learning state, and give interactive feedback (Lin et al., 2012; Hasan et al., 2020). A large volume of published studies has verified that ATS can improve learning motivation and outcomes (Lin et al., 2012; Sionti et al., 2018; Wang and Lin, 2018; Hasan et al., 2020). Particularly in language education, Ma and Lin (2017) discover that ATS can make Japanese learning more interesting and provide an adaptive learning environment. Similarly, Wu et al. (2022) note that the affective mobile language tutoring system (AMLTS) can deepen content understanding, promote engagement in peer interaction, and generate positive emotions. So far, ATS has been mainly applied in foreign language education, particularly in Japanese learning, whereas there are few studies on the use of ATS in the EFL context. Therefore, there is abundant room for further research exploring ATS's pedagogical and ethical implications in the EFL context and for further studies developing more effective applications utilizing AC to detect learners' emotions and proffer sufficient affective interaction and support in the EFL context.

## Learner-facing and teacher-facing AI in the EFL context

Aiming to present a more in-depth overview of AI in the EFL context, this review teased out published studies from two perspectives, namely learner-facing and teacher-facing AI applications (Baker and Smith, 2019). Generally, NMT tools, AI Chatbots and ITSs are related to learner-facing AI applications, which can promote adaptive or personalized learning, while AESs, IVE, and AC in ITSs can be concerned as teacher-facing systems, which can support teaching and reduce workload by automating administration, assessment, feedback, and data detection. However, in the authentic EFL context, some AI applications may play intertwined roles in promoting both teaching and learning. For instance, AESs, NMT tools, and ITSs can serve as monitoring and tutoring tools in the EFL instruction to promote EFL learning mainly from cognitive and linguistic perspectives (Groves and Mundt, 2015; Abu Ghali et al., 2018; Gao, 2021; Koh, 2022). EFL teachers can also harness IVE to construct an interactive and collaborative virtual reality learning environment to scaffold EFL learning (Melchor-Couto, 2017; Lan et al., 2018).

Additionally, it's noteworthy that the majority of published studies investigate EFL performance when applying AI in the EFL instructional settings, for instance, scaffolding argumentative writing by using AI chatbots (Guo et al., 2022), and improving speaking ability by deploying ITSs (Mohammadzadeh and Sarkhosh, 2018). Conversely, less research offer glimpse into the sheer EFL instruction behaviors from teachers' perspectives, although teachers' perceptions (e.g., willingness, attitude) of AI begin to draw attention (Sumakul et al., 2022). Similarly, teachers' consideration of ethical implications and risks when applying AI haven't been treated in much detail. These review results are in accordance with previous findings that there is a paucity of studies of AIEd from teachers'/teaching perspectives (Zawacki-Richter et al., 2019). Thus, more research is require to unravel AI's pedagogical potential to address muti-faceted EFL teaching issues (e.g., developing curriculum and materials, optimizing teaching modes, etc) and unfold new ethical implications and risks dwelling in AI inherently.

## Discussion

This review foregrounds that AI has empowered current EFL teaching and learning primarily in six forms and achieved relatively satisfactory effects and feedback, echoing related discussions (Sumakul et al., 2022), while some technical drawbacks and improper manipulation in authentic instruction still retain long-term conservative attention. Overall, the application of AESs, NMT Tools, ITSs, and AI Chatting Robots in the EFL context has made steady progress in promoting EFL teaching and learning, while IVE and AC in ITSs (e.g., ATS) still need to transcend current technical handicaps to fully spur their pedagogical potential and expand the breadth and depth of the application in the EFL context. Surprisingly, very few studies have explored AC in ITSs in EFL context. Moreover, other striking findings in this review are the dramatic paucity of empirical studies exploring pedagogical implications of AI in the EFL context from teachers' perspectives as well as less attention on ethical implications and risks when applying AI. Therefore, the following discussion will dig deeper into the challenges of AI in the EFL context from technical and teachers' perspectives and future research directions.

### Challenges from technical perspectives

With the deep learning (DL)-based AI techniques developing rapidly (Dong et al., 2021), DL has earned research attention in AIEd, especially in the EFL context. Traditional ML-based techniques mainly rely on experts' domain knowledge, whereby AI algorithms can be specifically designed for a given task, such as language recognition algorithm based on support vector machine (Campbell et al., 2004). In contrast, DL-based techniques are based on artificial neural network, e.g., convolutional neural network (Gu et al., 2018) and recurrent neural network (Mikolov et al., 2011), which can be easily adapted to handle different tasks. Moreover, multi-task learning methods have been developed using one DL model to cover different tasks (Dong et al., 2015), e.g., recognition and translation. However, DL-based techniques in existing AI-based EFL applications tend to analyze specific single signal, e.g., text for writing and audio for speaking. Accordingly, more severe challenges are arising from analyzing the multi-modal signals, namely text, audio, facial micro-expression and body actions, to promote the effects of EFL teaching and learning. To this end, large-scale Transformer (Shvetsova et al., 2022) can be pre-trained based on a large amount of multi-modal signals in self-supervised learning of multi-modal embedding space. For AI techniques in the EFL context, it is meaningful and challenging to adapt the large-scale multi-modal pre-training model to EFL teaching and learning.

This review also uncovers that existing AI-based EFL tools and systems are targeted to EFL performance and outcome from linguistic and cognitive perspectives, neglecting the emotions or mood during the learning process. In this vein, we suggest that affective computing (AC) is a potential solution to monitor the affective status of students to promote effectiveness in the EFL context (Lin et al., 2012; Hasan et al., 2020). In particular, students' emotions can be recognized and analyzed based on the physiological signal, e.g., audio, vision and even electroencephalo-graph, using AC techniques (Schoneveld et al., 2021), which can feed back to teachers or ITSs for further monitoring and improving the EFL learning. Considering the natural multi-modal property of physiological signals, the fusion of multi-type signals can also benefit from self-supervised learning in large-scale pre-training model but remains unresolved, when developing AC techniques for EFL fields. Besides aforementioned technical challenges, ethical issues of applying AC should be well addressed when collecting and analyzing multi-type physiological signals in a real-time manner to identify and measure learners' emotions during the entire learning process. In this sense, ethical implications were explored below from two perspectives, i.e., instructor and learner. On the one hand, AC should be properly used by the instructor for teaching (Wu et al., 2016a). Also when designing AI algorithms, AC for EFL should be strictly restricted to analyzing learning status, avoiding emotional manipulation or other commercial bias analysis. On the other hand, the learner's autonomy should be respected (Engel et al., 2017). For the private physiological signals, personal raw data should only be used in local personalized AI system, and cannot be uploaded to the public big data pool without authorization.

Personalized EFL tools and systems also require more focus, by which every student can be specifically guided by updating the DL model based on the learning process rather than sole English proficiency. Continual learning (Aljundi et al., 2019) can be adopted to adaptively update the DL model to satisfy personalized requirements and tasks in the EFL context. Continual learning and AC can also be integrated to adaptively finetune the DL model to explore both proficiency levels and emotional states in the EFL context. To this end, personalized EFL tools and systems based on Continual learning and AC would be the future direction, and they are undoubtedly facing head-on ethical challenges for avoiding the emotional manipulation, respecting autonomy, and protecting data privacy.

###  Challenges from teachers' perspectives

The review highlights that teachers' attitudes to applying AI in the EFL context tend to vary from being positive, which is in accord with Sumakul et al. (2022)'s findings, toward being conservative, as noted by Holstein et al. (2017) and Lin et al. (2017) that less-experienced instructors usually struggle to execute effective responses to analytics, leading to their reluctance and lower acceptance. Hence, from teachers' perspective, it's essential to relieve negative emotions and promote AI acceptance, aiming to deploy AI's pedagogical potential shored up by numerous empirical studies. Suggestions on improving AI acceptance *via* boosting confidence in applying AI and extending knowledge of AI are proffered and briefly explored below. Concretely, more future research foregrounded by ample empirical and theoretical evidence are still required to be conducted, aiming to highlight the effectiveness and potential of AI in the EFL context then galvanize confidence and motivation to utilize AI. Additionally, high-quality theoretical and practical profession training in AI can prepare teachers for AI-empowered EFL teaching, of which forms can be question-oriented practicums or workshops with modules of diverse EFL instructional contexts integrating multiple teaching modes rather than one-size-fits-all approach. It's noteworthy that improving teachers' awareness of ethical implications and risks when applying AI is compulsory (Russell, 2010). Finally, more access to engagement in interdisciplinary research with AI scholars can also provide technical support to EFL teachers, aiming to bolster the understanding and using of AI to maximize its pedagogical potential.

###  Future research directions

Notwithstanding a proliferation of studies on AI in the EFL context in recent years, there is a relative paucity of longitudinal studies investigating the effectiveness of AI in the EFL context *via* robust experiments with a larger amount of participants, strict assessment, competent instructors and supporting institutions, as well as a mechanism to protect data privacy. Besides, further research should be undertaken to investigate the adaptability of AI to different EFL learners with L2 individual differences (i.e., personal traits, language aptitude, motivation, learning styles, learning strategies) and to diverse learning and teaching contexts (e.g., online teaching, blended teaching, the flipped classroom). Additionally, there is abundant room for further progress in exploring students' emotional state by utilizing AC in the EFL context, hopefully contributing more evidence to detecting relationships between emotions and learning in the EFL context. Finally, future work is required to shed new lights on pedagogical implications as well as ethical implications and risks of AI in the EFL context.

## Author contributions

In the study, RJ was fully in charge of collecting and reviewing literature, writing, and revising the manuscript, etc.

## Funding

This work was supported by the 11th China Foreign Language Education Fund under Grant No. ZGWYJYJJ11A117.

## Conflict of interest

The author declares that the research was conducted in the absence of any commercial or financial relationships that could be construed as a potential conflict of interest.

## Publisher's note

All claims expressed in this article are solely those of the authors and do not necessarily represent those of their affiliated organizations, or those of the publisher, the editors and the reviewers. Any product that may be evaluated in this article, or claim that may be made by its manufacturer, is not guaranteed or endorsed by the publisher.
